# When Flexibility Is Stable: Implicit Long-Term Shaping of Olfactory Preferences

**DOI:** 10.1371/journal.pone.0037857

**Published:** 2012-06-21

**Authors:** Géraldine Coppin, Sylvain Delplanque, Christelle Porcherot, Isabelle Cayeux, David Sander

**Affiliations:** 1 Swiss Center for Affective Sciences, and the Laboratory for the Study of Emotion Elicitation and Expression (E3 Lab), Department of Psychology, University of Geneva, Geneva, Switzerland; 2 Firmenich SA, Geneva, Switzerland; CSIC-Univ Miguel Hernandez, Spain

## Abstract

Preferences are traditionally assumed to be stable. However, empirical evidence such as preference modulation following choices calls this assumption into question. The evolution of such postchoice preference over long time spans, even when choices have been explicitly forgotten, has so far not been studied. In two experiments, we investigated this question by using a variant of the free choice paradigm: In a first session, participants evaluated the pleasantness of a number of odors. We then formed pairs of similarly rated odors, and asked participants to choose their favorite, for each pair. Participants were then presented with all odors again, and asked for another pleasantness rating. In a second session 1 week later, a third pleasantness rating was obtained, and participants were again asked to choose between the same options. Results suggested postchoice preference modulation immediately and 1 week after choice for both chosen and rejected options, even when choices were not explicitly remembered. A third experiment, using another paradigm, confirmed that choice can have a modulatory impact on preferences, and that this modulation can be long-lasting. Taken together, these findings suggest that although preferences appear to be flexible because they are modulated by choices, this modulation also appears to be stable over time and even without explicit recollection of the choice. These results bring a new argument to the idea that postchoice preference modulation could rely on implicit mechanisms, and are consistent with the recent proposal that cognitive dissonance reduction could to some extent be implicit.

## Introduction

Preferences are typically assumed to be stable over time [1], [2] and are accordingly often described as relatively stable evaluations of stimuli in terms of pleasantness (see e.g., [3]). Despite long-standing research in psychology, neuroscience, and economics, understanding the sources and determinants of individual preferences remains a scientific challenge. Empirical evidence indicates that preferences are flexible in the sense that they could be modulated by cognitive variables such as familiarity [4], [5] and choices (e.g., [6], [7]). In the first experiment to address the question of how preferences could be modulated by choices, Brehm used the so-called *free-choice paradigm* and showed that just after a choice between two options evaluated as similarly desirable, that is, a difficult choice, participants rated the chosen option as more desirable and the rejected option as less desirable than they had rated them during the first evaluation.

Results from the free-choice paradigm have typically been explained by the cognitive dissonance reduction hypothesis [8]. In this context, cognitive dissonance corresponds to an aversive psychological state, which results from the conflict induced by the choice between the undesirable features of the chosen option and the desirable features of the rejected option [9], [10]. Cognitive dissonance reduction could be achieved by evaluating the chosen option more positively and the rejected option less positively. The reduction of cognitive dissonance is mostly thought to require conscious strategies and to be mediated by the accessibility of dissonant cognitions to one’s awareness [8], [11]–[16]. Consequently, this hypothesis requires an explicit memory of the choice [17], [18]. In addition to previous works having questioned awareness in cognitive dissonance (e.g., [19]–[21]), recent research has challenged the proposition that preference modulation following choice requires explicit memory. For instance, postchoice change in ranking have been shown in amnesic patients [18] whose brain lesion prevents them from remembering their choice, as well as in young children and capucin monkeys [22]. In healthy adult participants, Coppin and colleagues [7] have shown that pleasantness ratings were modified after choice, even when the choice was not explicitly remembered. The novel idea of their study was to use olfactory stimuli, which are known for evoking highly flexible responses [23], [24] and, in this respect, are particularly well suited to studying implicit memory [25]. Indeed, after the classical steps of the free-choice paradigm (ratings followed by choice, followed by new ratings), Coppin and colleagues [7] added an unexpected memory task about the choice made. The results suggested postchoice preference change in the sense of an increased evaluation of chosen odors and a decreased evaluation of rejected odors, even when choices were explicitly forgotten. Studying the influence of choice memory is relevant because it provides a more controlled basis for ruling out any explanation of postchoice pleasantness rating changes in terms of experimental demand, which has been identified as a critical bias when investigating postchoice preference modulation. Taken together, these results suggest that preference modulation following choice does not require explicit mechanisms. Instead, implicit mechanisms that are not necessarily accessible to conscious knowledge can apparently be sufficient to produce postchoice preference modulation.

Although the free-choice paradigm has been widely used during the last 50 years [26], to the best of our knowledge only a few previous studies have addressed the general issue of how stable the postchoice preference changes are over time. Frey and colleagues [27] have investigated postchoice attractiveness ratings modulation for books at four different time intervals (immediately, 3, 10, or 30 min after choice). They showed that the attractiveness ratings increase of chosen stimuli and the attractiveness ratings decrease of rejected stimuli (spreading of alternatives) were significant immediately after choice and stayed constant at the different time intervals included in this study. Walster [28] studied spreading of alternatives (jobs) immediately, 4, 15, and 90 min after choice. The results showed no postchoice attractiveness ratings change immediately after choice; increased attractiveness ratings for rejected stimuli and decreased attractiveness ratings for chosen stimuli attributed to postdecision regret after 4 min; increased attractiveness ratings for chosen stimuli and decreased attractiveness ratings for rejected stimuli after 15 min; and no attractiveness rating change 90 min after choice compared with before choice. Thus, the results of this study seem to indicate that postchoice attractiveness rating change is unstable over time and has already disappeared after 90 min. Few studies have investigated longer time spans (weeks or months) of postchoice pleasantness rating modulation for chosen stimuli [29]–[31]. Svenson and Benthorn [31] requested participants to make a choice between two alternatives of the same nature (e.g., stereos). Each of these alternatives was described in terms of four different attributes ranging from “very poor” to “very good” (e.g., price, design, sound quality, and overall quality of a set). Either immediately or 1 week later, participants were requested to judge how good or bad the alternatives were on the different characteristics. Their goal was consequently not to investigate the unfolding over time of pleasantness of *alternatives* as such, but of *characteristics* associated with alternatives. The results showed a significant increase in the quality ratings of the two most important attributes related to the chosen alternative after 1 week but not immediately after the decision. Gilbert and Ebert [29] showed an increased liking rating for a chosen stimulus (print), even 11 days after the choice, when participants did not have the opportunity to change their choice. To the contrary, Ritov [30] investigated the pleasure experienced with small gifts (desk organizer or paper holder) immediately and between 6 and 8 weeks after a choice varying in difficulty. The study showed a significant decrease of satisfaction rating with time. Vroom and Deci [32] found a very similar result regarding attitudes of graduate students towards the organization they chose to begin their career in: the attractiveness rating of the organization decreased during the first year after the choice, and stayed low during the next two and half years.

However, the results of all the above-mentioned studies have been recently rendered ambiguous. The methodology of the free-choice paradigm has indeed been under lively discussion recently, with a focus on what controls are appropriate when analyzing whether the choice phase truly causes a modulation in preferences (e.g., [33]–[38]).

Chen and Risen [34] provided a mathematical argument that under certain assumptions it is possible to measure postchoice preference change in the free-choice paradigm, even if the underlying preferences are actually stable. Sufficient assumptions to get this result are that (a) participants’ ratings are, at least partially, guided by their preferences; (b) participants’ choices are, at least partially, guided by their preferences (so that choices can reveal preferences); and (c) preferences cannot be perfectly measured by ratings. The authors formally derived that if these three assumptions hold, then the initial similarity of ratings between the chosen versus the rejected stimuli (that are presented in difficult pairs in the choice phase) may be due to errors in rating measures of an underlying stable “true” preference. The choice may then simply reveal which of the two had already initially been preferred. Consequently, during the second rating, one would also expect participants to rate the chosen stimuli as more pleasant and the rejected stimuli as less pleasant than during the first rating. This means that a measured postchoice preference change may not necessarily be driven by an influence of choice on preferences, but could also be an artifact caused by the way data are nonrandomly analyzed according to the choice, which reveals information about the underlying stable preferences. It is important to note that this criticism does not apply to other paradigms classically used to study cognitive dissonance, such as the effort-justification paradigm and the induced-compliance paradigm.

On the empirical side, Chen and Risen [34] suggested comparing the chosen spread in the classical rating-choice-rating (RCR) sequence with a control condition that modifies the sequence of measurements–a rating-rating-choice (RRC) condition–in which choice can reveal preferences but not influence the second rating. In a first empirical application, they could not find a difference in postchoice preference change between RCR and RRC. In a second study, the authors found a marginally significant difference (*p* = .06) between these sequences of measurement (RCR vs. RRC), suggesting the existence of choice-induced preference changes.

Recent works have addressed Chen and Risen’s [34] arguments both formally and empirically. One strand of work has scrutinized the formal basis of Chen and Risen’s argument [37]–[39], whereas another has provided empirical evidence that demonstrated an influence of choice on preferences despite Chen and Risen’s point [35], [36], [40].

On the theoretical side, Sagarin and Skrowonski [37], [38] argued that Chen and Risen’s argument “*rests on the unwarranted assumption that the…] choice provides a perfectly reliable measure of subjects’ preference for the chosen item over the unchosen item*”. They formally showed that the more random the choices, the less important Chen and Risen’s argument. How randomly choices reflect “true” preferences for similarly rated stimuli remains an empirical question–that is, the results derived with standard methodology are not necessarily rendered meaningless by Chen and Risen’s argument.

Moreover, two recent studies [35], [40] have empirically demonstrated postchoice preference modulation *when choice is manipulated*, thus establishing the effect in circumstances in which Chen and Risen’s argument does not seem to apply. Sharot et al. [40] used a “blind” choice, that is, a choice that participants *were not actually making*, even if they had the feeling that they did. Note that this strategy has also been recognized by Risen and Chen [41] as a valid approach to address the methodological issue under scrutiny. In such a paradigm, choice cannot be logically conceived as revealing anything about preference, yet results still showed reliable postchoice preference modulation. Furthermore, in a recent study conducted by Izuma et al. [36], postchoice preference change observed in RCR was shown to be significantly larger than preference change in RRC. The study thus demonstrates a postchoice preference modulation in terms of both ratings and brain activity levels, after controlling for the information revealed by choice. This empirical evidence suggests that Chen and Risen’s point should be taken into account in explaining a part of the effect that was previously exclusively attributed to postchoice modulation. Even if this effect of choice on preference is not as strong as previously thought, it still seems to be more than a mere artifact of the free choice paradigm, i.e. that there is a psychological influence of choice on preferences.

The present series of experiments emphasizes four aspects that were not included as the main focus in the studies previously described. The first and most crucial aspect is that in the research we report here, we added a control (Experiment 3) for this issue raised by Chen and Risen. This control confirms the robustness of postchoice preference change to settings in which Chen and Risen’s [34] point does not apply. The second aspect is the investigation of postchoice pleasantness rating modulation across long time spans for chosen, but also for rejected, alternatives. The third aspect is the assessment of *experienced utility* [42], that is, the subjective pleasure experienced with a stimulus, and not the *predicted utility,* that is, the beliefs about the subjective pleasure experience with a stimulus. In Ritov’s study [30], participants were asked by phone to assess the pleasure of the chosen option. However, this method might not be the optimal way to study long-term preference evolution over time. Indeed, such a question does not allow an optimal assessment of experienced utility [42]. An advantage of actually presenting olfactory stimuli in the series of experiments is that it directly impacts experienced utility. The fourth aspect emphasized by our studies is the investigation of the role of choice memory for postchoice pleasantness rating modulation across long time spans. Indeed, no study to date has considered whether postchoice pleasasantness rating modulation is stable over time when choices are forgotten. However, studying the influence of choice memory is relevant because it provides a more controlled basis for ruling out any explanation of postchoice pleasantness rating changes in terms of experimental demand. According to impression management theory [43], participants would want to give a good impression of themselves to the experimenter and might then report evaluations consistent with the choices they had previously made, even if such evaluations were not genuine. The study designed here addresses this matter because such a motivation is difficult to conceive in the absence of an explicit memory of the choice.

In summary, no study, to the best of our knowledge, has thoroughly rejected the possibility that postchoice preference modulation–*for both chosen and rejected stimuli* and *during the experienced pleasantness elicited by an actual presentation of these stimuli*–is not an epiphenomenon, fading away after some time has passed. Crucially, no study so far have investigated postchoice preference modulation in long time spans in controlling that this effect is not an artifact of the free-choice paradigm [34]. Moreover, even if postchoice preference modulation appears stable over time when the choice is explicitly remembered, one could not rule out an explanation in terms of experimental demands. A stronger test of the hypothesis that postchoice preference modulation appears stable over time is therefore to test whether the effect is still present after a long delay even when the choice itself has been forgotten. This question was also address here by experimentally investigating the long-term evolution of postchoice olfactory preferences over time, even when choices themselves are forgotten.

We conducted a series of three experiments in order to address these questions. Specifically, in the first two experiments, using an adapted version of the free-choice paradigm, we tested whether postchoice preference modulation can be robust for a 1-week interval and whether results vary depending on participants’ explicit memory of their initial choices. In the third experiment, using a paradigm addressing recent criticisms regarding the true impact of choices on preferences [34], we aimed at confirming that choice can have a modulatory impact on preferences, and that this modulation can be long-lasting. Those aims were achieved.

## Experiment 1

### Method

#### Ethics statement

This first experiment, as well as Experiments 2 and 3, part of the EmOdor project, have been approved by the ethics committee of the Faculty of Psychology and Sciences of Education (FPSE) at the University of Geneva. All participants were over 18 and gave a written consent form.

#### Participants

Forty-one University of Geneva students (33 females, 8 males; mean age = 24.51±7.53 years) took part in this experiment. Before starting the experiment, participants completed a written consent form. All participants reported a normal sense of smell. They were individually tested and were paid 15 Swiss Francs (about 15 US Dollars) for their participation. During the days of testing, they were asked not to wear any fragrance.

#### Stimuli

Sixteen odorants (provided by Firmenich, SA) were selected on the basis of their ratings of pleasantness, familiarity, and intensity obtained from previous studies [4], [7], [44]. To hinder odor recognition, we excluded very familiar odors because of the probability of their being easily recognized [45]. We also excluded odors that were extreme in valence or intensity. The mean ratings of the selected odors are provided in Table 1. Odorants were diluted in odorless dipropylene glycol to obtain a roughly similar mean intensity (see [4], for further details). Solutions (4 ml) were injected into the absorbent core of cylindrical felt-tip pens (14 cm long, inner diameter 1.3 cm). The use of these devices (provided by Burghart, Germany) avoids any olfactory contamination of the environment. Each odorant was coded by a random three-digit code.

**Table 1:** Names of the Eight Target Odors Used in Study 1 and Their Mean Ratings.

Odor	Mean pleasantnessbefore choice	Mean pleasantnessafter choice	Mean pleasantnessafter choice(1 week later)	Mean familiaritybefore choice	Mean familiarity after choice	Mean familiarityafter choice (1 week later)	Mean intensitybefore choice	Mean intensityafter choice	Mean intensityafter choice (1week later)
Detergent	6.15 (±2.07)	6.63 (±1.90)	6.83 (±2.05)	7.09 (±2.14)	6.96 (±1.86)	6.95 (±2.17)	4.67 (±2.36)	4.65 (±2.53)	4.69 (±2.21)
Shampoo fragrance	7.55 (±1.91)	7.83 (±1.39)	7.88 (±1.33)	7.17 (±2.06)	7.68 (±1.69)	7.42 (±1.86)	6.30 (±1.90)	6.18 (±2.13)	6.22 (±1.76)
Fig flower	5.49 (±2.07)	4.57 (±2.49)	4.95 (±2.17)	5.76 (±2.69)	5.72 (±2.53)	5.38 (±2.42)	6.04 (±1.89)	6.78 (±1.71)	6.51 (±1.73)
Lilac flower	6.41 (±2.44)	6.08 (±1.95)	6.17 (±2.09)	6.76 (±2.20)	6.61 (±2.01)	6.80 (±1.87)	6.61 (±1.96)	6.38 (±1.59)	6.33 (±1.53)
Violet flower	5.07 (±1.95)	4.73 (±1.75)	5.33 (±1.68)	4.35 (±2.33)	4.69 (±2.05)	4.92 (±2.49)	5.29 (±2.25)	5.22 (±2.31)	5.59 (±2.18)
Yoghurt	3.19 (±1.57)	3.01 (±1.95)	2.77 (±1.82)	6.12 (±2.61)	5.95 (±2.47)	6.04 (±2.31)	7.48 (±1.54)	7.57 (±1.67)	7.74 (±1.48)
Aladinate (floral note)	3.49 (±2.10)	3.37 (±1.85)	3.22 (±1.54)	4.80 (±2.71)	4.67 (±2.39)	4.49 (±2.31)	7.28 (±1.75)	7.47 (±1.81)	7.48 (±1.25)
Melon	3.54 (±1.66)	3.16 (±1.95)	3.27 (±1.71)	4.25 (±2.56)	4.14 (±2.23)	5.02 (±2.41)	7.14 (±1.58)	7.36 (±1.86)	7.39 (±1.61)

*Note*. Standard deviations are presented in parentheses.

#### Procedure

The present experiment was divided into two parts, separated by 1 week. The time of day of the experimental session was unchanged for a given participant.

#### First session

First, we assessed the individual’s ratings of pleasantness, familiarity, and intensity for 8 of the 16 odors (target odors). On the basis of these first pleasantness ratings, four pairs were created for the choice phase. During this choice phase, participants were presented with two kinds of odor pairs: (a) two pairs of odors that they had rated as very similarly pleasant (i.e., difficult choice conditions for one half of the trials; mean rating differences = 0.31±0.39 on the 10-point subjective scale described in the following paragraph), and (b) two pairs of odors that they had rated very differently for pleasantness (i.e., easy choice conditions for one half of the trials; mean rating differences = 3.07±1.28 on the 10-point subjective scale described in the following paragraph). Second, for each pair, participants were required to choose the odor they preferred. Third, after these choices had been made, participants were again requested to assess the pleasantness, familiarity, and intensity of the eight odors (see Table 1). Finally, participants were presented with the eight already presented target odors, together with eight new odors (distractors) and were requested to indicate whether they had already smelled each odor during the experiment. For each odor, if they answered yes, they were then asked to specify if they had designated this odor as chosen or rejected during the choice phase. Before being asked to do so at the end of the experiment, participants were not aware that they would have to complete this memory task. During the entire experiment, the order in which odors or pairs of odors were presented was controlled. For each presentation of the odor, participants were instructed to smell the odor during two inspirations at most.

#### Second session 1 week later

First, participants were asked to assess the pleasantness of the eight odorants already used during the first session. Second, they were asked to choose the odor they preferred for the same pairs of odors that they were presented with during the first session, 1 week before. These pairs were specific to a given participant, as they were created during the first session on the basis of participant’s first pleasantness ratings.

#### Subjective ratings for the two sessions

After each odorant presentation (except during the choice phases), participants were asked to rate the pleasantness, familiarity, and intensity on continuous scales presented on a computer screen. Participants had to move a vertical marker with the mouse across a horizontal line and click to indicate their rating. Participants judged the pleasantness of the odor from *very unpleasant* (left on the scale = 1) to *very pleasant* (right on the scale = 10); the subjective familiarity from *not familiar at all* (left) to *very familiar* (right); and the subjective intensity from *not perceived* (left) to *very strong* (right).

#### Data analyses

The difference between prechoice and postchoice ratings was calculated for each of the eight target odors. For each participant, we assessed odor recognition memory performance by using parameters based on signal detection theory [46]. If the odor was presented during the experiment and declared so by a subject, a “hit” was scored. If the odor was not presented during the experiment but declared so, a “false alarm” was recorded. From hit and false-alarm scores, we then calculated four parameters: hit rate (HR), false-alarm rate (FR), discrimination rate (d’L), and response bias (CL). We also assessed the memory performance as a function of the choice by using the same procedure, with a “hit” being recorded if the odor was chosen or rejected and the participant declared so accurately. For the analyses performed on the subjective ratings (pleasantness, intensity, and familiarity), we defined a trial as remembered if the participant correctly recalled the choice he or she made. Otherwise, the trial was considered as forgotten.

### Results

#### Pleasantness Changes Following Choice

Choice-induced changes are typically reported when the choice is difficult, that is, in this case, when the difference between the pleasantness ratings of the two paired odors obtained before the choices is small. We performed a repeated measures ANOVA with the factors period (before choice, after choice) and choice (chosen, rejected) on the pleasantness scores in the difficult-choice condition. The interaction between these factors was significant [*F*(1, 40) = 21.94, *p*<.001, η^2^ = .35], revealing the classical effect of pleasantness rating modulation after choice. Pleasantness ratings were significantly decreased for rejected odors [*F*(1,40) = 5.40, *p* = .025, η^2^ = .12], and pleasantness ratings were significantly increased for chosen odors [*F*(1,40) = 10.27, *p* = .003, η^2^ = .20]. An identical analysis performed on the pleasantness scores in the easy-choice condition revealed only a significant main effect of choice [*F*(1, 40) = 100.18, *p*<.001, η^2^ = .71], which reflected significantly higher pleasantness ratings for chosen odors than for rejected ones.

#### Pleasantness Changes Following Choice during the Second Session (After 1 Week)

**Figure 1:**
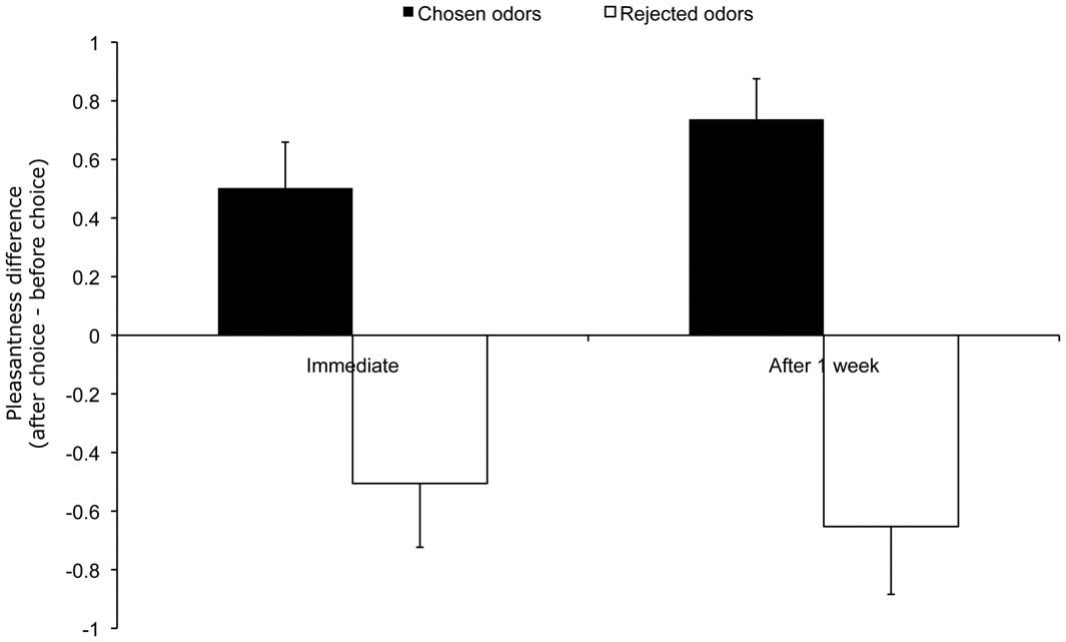
Pleasantness rating modulation following choice between two very similarly pleasant odors in Experiment 1. The bars on the left represent the difference between pleasantness ratings before and just after choosing between pairs of odors. The bars on the right represent the difference between pleasantness ratings before and 1 week after these choices. Values greater than 0 indicate an increase in pleasantness ratings for the odor after choice and values less than 0 a decrease in pleasantness ratings. Error bars represent the standard error to the mean.

We performed a repeated measures ANOVA with the factors period (before choice, 1 week after choice) and choice (chosen, rejected) on the pleasantness scores in the difficult-choice condition. Results were similar to those obtained just after the choice: the interaction between these factors was significant [*F*(1, 40) = 31.42, *p*<.001, η^2^ = .44]. This result critically indicates that pleasantness rating modulation was still present 1 week after the choice (see figure 1). Pleasantness ratings decreased significantly for rejected odors [*F*(1,40) = 7.94, *p* = .007, η^2^ = .17] and pleasantness ratings increased significantly for chosen odors [*F*(1,40) = 28.40, *p*<.001, η^2^ = .42].

The repeated measures ANOVA with the factors period (immediately after choice, 1 week after choice) and choice (chosen, rejected) performed on the difference between prechoice and postchoice ratings revealed no significant interaction between these factors [*F*(1,40) = 1.65, p = .206]. Taken together, these results indicate that pleasantness rating changes were not statistically different immediately after and 1 week after the choice, which argues for stability over time.

In the easy-choice condition, the repeated measures ANOVA with the factors period (before choice, one week after choice) and choice (chosen, rejected) on the pleasantness scores only revealed a significant main effect of choice [*F*(1, 40 = 96.30, *p*<.001, η^2^ = .71], which reflected significantly higher pleasantness ratings for chosen odors than for rejected ones.

The following analyses are conducted for the difficult-choice condition only.

#### Memory performance

We first assessed odor recognition memory performance. As indicated by the mean hit rate, discrimination rate, and response bias (HR = 0.90, d’L = 4.76, CL = −0.03), participants remembered and well discriminated the presented odors from the distracting odors. Memory performance for the choice remains intermediate (HR = 0.63), even though it was significantly above chance (*t* test for single mean: *t*(40) = 5.43, *p*<.001).

#### Role of the explicit memory of the choice

Only 7 of the 41 participants had at least one rejected and one chosen odor for both forgotten and remembered conditions. Thus, we were unable to robustly test whether pleasantness rating change demonstrated immediately after choices differed from those demonstrated after 1 week, according to whether choices were remembered or not. Apparently, the memory task was not difficult enough for the participants. Both the fact that the number of target and distractive odors was small and the fact that they were distributed in equal proportion (eight odors for both categories) did not favor forgetting.

#### Influence of pleasantness on choices

We checked whether the pleasantness of the odor before the choice varied as a function of the participant’s choice. A repeated measures ANOVA with the factor choice (chosen, rejected) performed on pleasantness scores before the choice was marginally significant [*F*(1,40) = 3.39, *p* = .078].

#### Congruency between choices

The likelihood of making the same choice (either chosen or rejected) again in the second session was far above chance [forgotten condition: *t* test for single mean: *t*(38) = 5.33, *p*<.001; remembered condition: *t* test for single mean: *t*(39) = 7.16, *p*<.001], independently of whether participants remembered which smell they chose 1 week earlier (80%) or not (79%) [*F*(1,35) = 0.10, p = .758].

#### Familiarity and intensity ratings

The mean familiarity and intensity ratings for all the odors are reported in Table 1. There was neither a difference between the familiarity ratings of the chosen and the rejected odors prior to difficult choices, [*F*(1,40) = 2.46, *p* = .124] nor between the intensity ratings [*F*(1,40) = 0.46, p = .502]. It is then very unlikely that choices could have been based on a priori differences in the familiarity or intensity ratings of the paired odors. There was a statistical difference in familiarity ratings between chosen and rejected odors immediately after the first choice [*F*(1,40 = 4.75, *p* = .035], and a statistical trend 1 week later [*F*(1,40) = 3.47, p = .070]. There was neither a difference in intensity ratings immediately after the choice [*F*(1,40) = 0.51, p = .481] nor 1 week later [*F*(1,40 = 0.71, p = .403]. Thus, the repetition of odor presentation did not seem to modify the intensity ratings.

### Discussion

These data suggest that olfactory pleasantness ratings could be modulated by a difficult choice in long time spans. Indeed, both 10 min and 1 week after a difficult choice, chosen odors were valuated more positively and rejected odors were valuated less positively compared with a first pleasantness evaluation. Thus, in addition to replicating a long time span pleasantness rating modulation for chosen stimuli, this study also shows such a modulation for rejected stimuli. Furthermore, the results reveal that postchoice pleasantness rating modulation is also present when measuring for experienced utility and not only for predicted utility. Moreover, the coherence of choices over time was high, even when choices were not explicitly remembered.

In contrast to a previous experiment [7] in which six pairs of odors were presented, this study only included four pairs of odors. Consequently, the difficulty of the task in terms of memory was not high enough to allow a reliable comparison between chosen and rejected odors in both forgotten and remembered choices, as remembered choices were much more numerous than forgotten ones. Hence, we decided to conduct a second experiment in which we increased the number of pairs presented to increase the difficulty in terms of memory and consequently to be able to compare conditions in which choices were forgotten and those in which choices were remembered.

## Experiment 2

### Method

#### Participants

Thirty-five University of Geneva students (30 females, 5 males; mean age = 22.41±4.97 years) took part in this experiment. Before starting the experiment, participants completed a written consent form. All participants reported a normal sense of smell. They were individually tested and asked not to wear any fragrance during the days of testing.

#### Stimuli

Twenty-six odors were used in this experiment. Their mean ratings are provided in Table 2.

**Table 2:** Names of the 16 Target Odors Used in Study 2 and Their Mean Ratings.

Odor	Mean pleasantnessbefore choice	Mean pleasantnessafter choice	Mean pleasantness after choice (1 week later)	Mean familiarity before choice	Mean familiarity after choice	Mean familiarity after choice (1 week later)	Mean intensity before choice	Mean intensity after choice	Mean intensity after choice (1 week later)
Detergent	5.47 (±1.90)	5.80 (±1.85)	5.65 (±1.83)	6.00 (±2.77)	6.01 (±2.20)	5.93 (±2.42)	4.47 (±2.00)	3.98 (±2.31)	3.68 (±2.29)
Shampoo fragrance	7.69 (±1.38)	7.34 (±1.90)	7.70 (±1.41)	7.30 (±2.03)	7.59 (±2.11)	7.35 (±2.29)	6.16 (±1.93)	5.99 (±1.89)	6.17 (±1.88)
Fig flower	5.75 (±2.15)	5.32 (±2.37)	5.76 (±2.12)	5.21 (±2.81)	5.25 (±2.20)	5.37 (±2.54)	6.16 (±1.81)	6.47 (±1.41)	6.12 (±1.82)
Lilac flower	6.57 (±2.10)	6.80 (±1.65)	6.97 (±1.88)	6.92 (±2.08)	6.35 (±2.04)	6.22 (±2.05)	5.83 (±2.06)	5.51 (±1.95)	4.79 (±2.23)
Violet flower	5.26 (±1.82)	5.84 (±2.12)	5.68 (±2.08)	4.06 (±2.23)	4.54 (±1.97)	4.63 (±2.32)	4.53 (±1.95)	5.06 (±2.13)	4.72 (±2.50)
Yoghurt	2.84 (±2.03)	2.86 (±1.89)	3.00 (±1.88)	5.56 (±2.85)	5.41 (±2.62)	5.05 (±2.36)	6.76 (±2.10)	7.48 (±1.86)	6.91 (±1.93)
Aladinate (floral note)	2.97 (±2.18)	3.34 (±2.08)	3.43 (±1.92)	4.20 (±2.46)	4.36 (±2.51)	4.74 (±2.42)	6.76 (±1.99)	7.00 (±1.75)	6.66 (±1.60)
Melon	2.96 (±1.91)	2.70 (±1.97)	3.27 (±1.55)	3.23 (±2.34)	3.90 (±2.40)	4.36 (±2.17)	7.21 (±1.81)	7.53 (±1.62)	7.24 (±1.45)
Sandalwood	4.25 (±1.71)	4.17 (±1.93)	4.18 (±1.70)	4.41 (±2.33)	5.18 (±2.21)	5.13 (±2.29)	5.23 (±1.86)	5.81 (±1.85)	5.23 (±2.14)
Lavender flower	6.09 (±2.75)	6.21 (±2.60)	6.48 (±2.42)	7.34 (±2.19)	7.36 (±2.13)	7.50 (±2.18)	7.04 (±1.55)	7.46 (±1.75)	7.20 (±1.62)
Raspberry flower	6.24 (±1.68)	5.90 (±1.65)	6.21 (±1.92)	4.34 (±2.24)	4.38 (±1.90)	4.03 (±2.20)	3.41 (±1.92)	2.78 (±2.23)	3.66 (±2.41)
Tutti fruiti	6.96 (±2.41)	6.94 (±2.19)	7.18 (±2.14)	8.38 (±1.63)	8.49 (±1.33)	8.43 (±1.34)	7.26 (±1.56)	7.51 (±1.21)	7.40 (±1.34)
Pineapple	7.14 (±1.63)	6.84 (±1.83)	7.48 (±1.38)	7.22 (±1.78)	6.80 (±1.88)	6.88 (±2.01)	6.47 (±1.43)	6.41 (±1.57)	6.18 (±1.79)
Magnolia	5.96 (±1.94)	5.73 (±1.77)	6.02 (±1.71)	4.84 (±2.27)	5.18 (±2.29)	4.22 (±2.27)	6.47 (±1.57)	6.26 (±1.85)	6.06 (±1.85)
Beer	2.98 (±1.65)	3.30 (±2.16)	2.93 (±1.73)	4.93 (±2.94)	5.58 (±2.79)	5.68 (±2.48)	6.80 (±1.55)	6.70 (±1.29)	7.21 (±1.81)
Leather	2.55 (±1.73)	3.11 (±2.05)	3.02 (±1.93)	3.77 (±2.92)	4.63 (±2.75)	4.98 (±2.36)	6.00 (±2.09)	6.44 (±1.84)	6.60 (±1.64)

*Note*. Standard deviations are presented in parentheses.

#### Procedure

The procedure was similar to the one used in Study 1. The major difference was that 16 odors were used as targets here, and consequently 8 pairs of odors were created during the choice session (instead of 4 as in Study 1). Thus, during this session, participants were presented with two kinds of odor pairs: (a) four pairs of odors that they had rated as similarly pleasant (i.e., difficult choice conditions for one half of the trials; mean rating differences = 0.10±0.09 on the 10-point subjective scale) and (b) four pairs of odors that they had rated differently for pleasantness (i.e., easy choice conditions for one half of the trials; mean rating differences = 3.56±2.55 on the 10-point subjective scale). Another slight adaptation from Study 1 was that 10 odors were used as distractors (rather than 8 as in Study 1).

### Results

#### Pleasantness changes following choice

We performed a repeated measures ANOVA with the factors period (before choice, after choice) and choice (chosen, rejected) on the pleasantness scores in the difficult-choice condition. The interaction between these factors was significant [*F*(1,34) = 29.95, *p*<.001, η^2^ = .47], showing again the well-documented effect of pleasantness rating change following difficult choices. Pleasantness ratings were significantly increased for chosen odors [*F*(1,34) = 29.63, *p*<.001, η^2^ = .47]; the decrease in pleasantness ratings for rejected odors was not significant [*F*(1,34) = 2.71, *p* = .109]. An identical analysis performed on the pleasantness scores in the easy-choice condition revealed only a significant main effect of choice [*F*(1, 34 = 131.73, *p*<.001, η^2^ = .79], which reflected significantly higher pleasantness ratings for chosen odors than for rejected ones.

#### Pleasantness changes following choices during the second session (after 1 week)

The same analyses were performed 1 week after choice. The repeated measures ANOVA with the factors period (before choice, 1 week after choice) and choice (chosen, rejected) performed on the pleasantness scores was significant [*F*(1,34) = 18.02, *p*<.001, η^2^ = .35]. This result indicates that pleasantness rating modulation was still present 1 week after the choice. Pleasantness ratings were significantly increased for chosen odors [*F*(1,34) = 16.06, *p*<.001, η^2^ = .32]; the decrease in pleasantness ratings for rejected odors did not reach significance [*F*(1,34) = 1.19, p = .283].

The repeated measures ANOVA with the factors period (immediately after choice, 1 week after choice) and choice (chosen, rejected) performed on the difference between prechoice and postchoice ratings revealed no significant interaction between these factors [*F*(1,34) = 0.09, p = .767]. Taken together, these results indicate that pleasantness ratings changes were not statistically different immediately after and 1 week after the choice, which suggests stability over time.

In the easy-choice condition, the repeated measures ANOVA with the factor period (before choice, one week after choice) and choice (chosen, rejected) on the pleasantness scores only revealed a main effect of choice [*F*(1, 34)\ = 139.47, *p*<.001, η^2^ = .80], which reflected significantly higher pleasantness ratings for chosen odors than for rejected ones.

The following analyses are conducted for the difficult-choice condition only.

#### Memory performance

As in Study 1, participants remembered and well discriminated the presented odors from the distracting odors (mean hit rate: HR = 0.85, d’L = 3.46, CL = −0.27). Memory performance for the choice was again intermediate (R = 0.60), even if still significantly above chance (*t* test for single mean: *t*(35) = 4.306, *p*<.001).

#### Role of explicit memory of the choice during the first session

**Figure 2:**
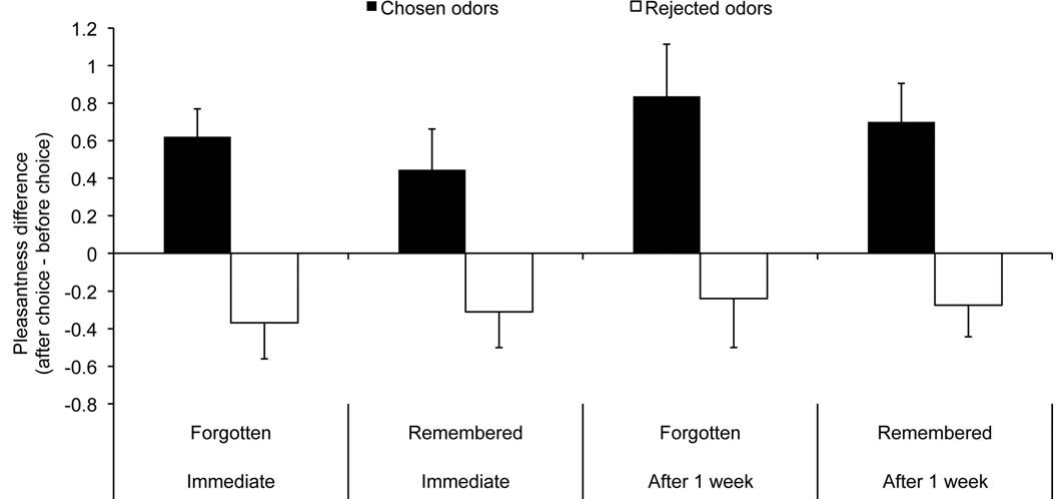
Pleasantness rating modulation following choice between two very similarly pleasant odors in Experiment 2. The bars on the left represent the difference between pleasantness ratings before and just after choosing between pairs of odors, for both forgotten and remembered choices. The bars on the right represent the difference between pleasantness ratings before and 1 week after these choices, again for both forgotten and remembered choices. Values greater than 0 indicate an increase in pleasantness ratings for the odor after choice and values less than 0 a decrease in pleasantness ratings. Error bars represent the standard error to the mean.

To assess the role of memory for the choice in pleasantness rating change, a repeated measures ANOVA with the factors choice (chosen, rejected) and memory (remembered, forgotten) was performed on the difference score in the difficult-choice condition (after choice - before choice). This analysis revealed a main effect only of choice, [*F*(1,20) = 7.68, *p* = .012, η^2^ = .28], showing that the difference between the pleasantness evaluation increase of the chosen odors and the pleasantness evaluation decrease of the rejected odors was significant for forgotten and remembered choices combined. Two repeated measures ANOVAs conducted on the difference scores for the forgotten and remembered difficult choices separately confirmed significant postchoice pleasantness rating change for remembered choices, but critically, also for forgotten choices [*F*(1,28) = 5.48, *p* = .027, η^2^ = .16 and *F*(1,24) = 8.29, *p* = .008, η^2^ = .26, respectively] (see figure 2).

#### Role of explicit memory of the choice after 1 week

We performed a repeated measures ANOVA with the factors choice (chosen, rejected) and memory (remembered, forgotten) on the difference scores in the difficult-choice condition (1 week after choice – before choice). This analysis revealed a main effect of choice [*F*(1,20) = 7.54, *p* = .012, η^2^ = .27]. Two repeated measures ANOVAs conducted on the difference scores for the forgotten and remembered difficult choices separately confirmed significant postchoice pleasantness rating change for remembered choices, but critically, also for forgotten choices [*F*(1,28) = 13.96, *p*<.001, η^2^ = .33 and *F*(1,24) = 5.35, *p* = .030, η^2^ = .18, respectively] (see figure 2).

#### Congruency between choices

The likelihood of making the same choice again in the second session was far above chance [forgotten condition: *t* test for single mean: *t*(35) = 6.29, *p*<.001; remembered condition: *t* test for single mean: *t*(35) = 7.04, *p*<.001], independently of whether participants remembered which smell they chose 1 week earlier (82%) or not (79%) [F(1,35) = 0.49, p = .487].

#### Influence of pleasantness on choices

We checked whether the pleasantness of the odor before the choice varied as a function of the participant’s choice. The repeated measures ANOVA with the factor choice (chosen, rejected) performed on pleasantness scores before the choice was marginally significant [*F*(1,34) = 3.92, *p* = .056] – odors evaluated as the most pleasant before choice tend to be chosen during the choice phase.

#### Familiarity and intensity ratings

The mean familiarity and intensity ratings for all the odors are reported in Table 2.

There was a difference neither between the familiarity ratings of the chosen and the rejected odors prior to difficult choices [*F*(1,34) = 0.04, p = .853], nor between the intensity ratings [*F*(1,34) = 1.26, p = .270]. Familiarity ratings between chosen and rejected odors were not significantly different either just after the first choice [*F*(1,34) = 0.11, p = .738] or 1 week after the first choice [*F*(1,34) = 0.13, p = .717]. Intensity ratings between chosen and rejected odors were marginally significantly different just after the first choice [*F*(1,34) = 3.70, *p* = .062] and significantly different 1 week after the first choice [*F*(1,34) = 5.21, *p* = .029, η^2^ = .13], rejected odors being rated on average as more intense than chosen odors.

As in Study 1, following a difficult choice, pleasantness ratings were modulated–chosen odors were valuated more positively compared with a first pleasantness evaluation–both immediately after choice and also 1 week later. However, in contrast to the findings of Study 1, the more negative evaluation of rejected odors, compared with a first pleasantness evaluation, was did not reach significance immediately after choice (statistical trend) and 1 week later. As the mean ratings of pleasantness before choice were very similar for both experiments (mean pleasantness ratings before choice for Experiment 1 = 5.11±1.59 and for Experiment 2 = 5.11±1.75), these findings cannot be explained by an overall differential attractiveness of the stimuli in the two experiments [47]. Previous research has also demonstrated devaluations of odors after their rejection [7].

Pleasantness ratings before choice tend to predict participants’ choices: odors evaluated as the most pleasant before choice tend to also be the chosen ones. This result tends to confirm one of Chen and Risen [34]’ assumptions, i.e. participants’ choices are, at least partially, guided by their preferences. Choices could consequently reveal preferences.

Increasing the number of odorants had the predicted effect: The number of remembered and forgotten choices were more balanced than in Experiment 1, allowing for a statistical comparison of the impact of choice memory on postchoice pleasantness rating modulation in both the first and the second sessions. Following difficult choices, chosen odors were evaluated more positively and rejected odors less positively, even when choices were not explicitly remembered. This result provides support for the hypothesis that implicit mechanisms underlie the observed pleasantness rating modulation with long-term stability.

However, as described in the introduction, we cannot unequivocally conclude from Experiments 1 and 2, as based on the free-choice paradigm [34], that choice has a modulatory impact on preferences.

To check the validity of the conclusions we drew from Experiments 1 and 2, i.e. that choice has a modulatory impact on preferences and that this modulation is long-lasting, we conducted a third experiment. The design of this experiment was adapted from the effort-justification paradigm [48], where Chen and Risen’s rationale does not apply. Furthermore, Chen and Risen’s point has no bearing on our third conclusion from Experiments 1 and 2, i.e. that postchoice preference modulation does not require an explicit memory of the choice.

As mentioned above, the effort-justification paradigm does not suffer from the point raised by Chen and Risen (see [41]). This paradigm aims at experimentally demonstrating that “*persons who go through a great deal of trouble or pain to attain something tend value it more highly than persons who attain the same thing with a minimum of effort*” (Aronson & Mills, 1959, p.177). In Aronson & Mills’ princeps study [48], participants went through either a mild or severe initiation to join a group. Importantly, even if participants had the feeling they were choosing to go through the initiation or not, they actually did not – they were assigned randomly to one of the two initiation groups. They did not choose either which type of initiation they were going through. This is precisely because going through a mild or severe initiation did not reveal participants’ pre-existing preference for the group that the effort-justification paradigm is valid [41]. After the initiation, the group they went through the initiation for turned out to be dull. Participants were asked to fill in 17 rating scales regarding the group. These ratings were averaged to provide a group liking measure. Participants who went through a severe initiation liked the group more than participants in the mild initiation condition. This severe initiation condition is indeed supposed to raise cognitive dissonance [48].

We adapted this idea to our setting by adding a choice phase, in a way that could not possibly reflect participants’ preferences for the odors presented. Specifically, participants were asked to decide, before each odor presentation, whether they wanted to spend money on this trial or not. Due to the restriction that they had to pay on exactly half the trials, participants effectively chose *for which* trials they wanted to sacrifice money. Crucially, since the monetary choice was made *before* smelling the odor, this choice cannot, by definition, reflect the participant’s preferences. We subsequently measured how this choice affected pleasantness’ ratings of the odors both immediately and one week after choice. The odors could be anywhere on the pleasantness spectrum.

The hypothesis behind this design is that in cases where participants pay for what turns out to be a bad odor, this will cause cognitive dissonance. Thus, we expected that bad odors would be rated *less unpleasant when paid for*, compared to when they were not paid for.

## Experiment 3

### Method

#### Participants

Twenty-six participants were tested in the experiment after completing a written consent form. We lost the data of 3 of them because of technical problems, leaving the data from 23 participants usable (20 females, mean age = 23.52, s.d. = 4.58). All participants reported a normal sense of smell. They were individually tested and were asked not to wear any fragrance during the days of testing. They were paid 12 Swiss francs (about 12 US Dollars) for their participation.

#### Stimuli

Thirty odors were used in this experiment. Their mean ratings are provided in Table 3. Odorants were diluted in odorless dipropylene glycol to obtain a roughly similar mean intensity [see 4 for further details]. Solutions (4 ml) were injected into the absorbent core of cylindrical felt-tip pens (14 cm long, inner diameter 1.3 cm). The use of these devices (provided by Burghart, Germany) avoids any olfactory contamination of the environment. Each odorant was coded by a random three-digit code.

**Table 3:** Names of the Twenty Odors Used in Study 3 and Their Mean Ratings.

Odor	Mean pleasantnessbefore choice	Mean pleasantnessafter choice	Mean pleasantness after choice(1 week later)	Mean familiarity before choice	Mean familiarity after choice	Mean familiarity after choice (1 week later)	Mean intensity before choice	Mean intensity after choice	Mean intensity after choice (1 week later)
Aladinate (Floral note)	4.39 (±2.80)	4.77 (±2.75)	4.93 (±2.42)	4.39 (±2.65)	4.90 (±2.35)	4.74 (±1.97)	7.46 (±1.68)	7.68 (±1.55)	7.53 (±2.02)
Mint	5.52 (±2.02)	6.30 (±2.26)	6.13 (±2.53)	7.52 (±2.24)	7.87 (±1.84)	7.77 (±2.64)	6.90 (±1.43)	6.85 (±1.64)	7.12 (±1.55)
Sandalwood	3.08 (±1.97)	2.88 (±1.41)	2.93 (±1.99)	3.74 (±3.13)	3.82 (±2.21)	4.32 (±2.24)	5.73 (±2.42)	6.44 (±1.57)	6.43 (±1.96)
Orange	6.47 (±2.00)	7.23 (±1.77)	6.79 (±1.98)	6.73 (±2.13)	7.11 (±1.97)	6.59 (±2.35)	6.63 (±1.57)	6.46 (±1.27)	6.51 (±1.71)
Lavender	5.19 (±2.40)	5.01 (±1.95)	5.52 (±2.18)	5.56 (±2.46)	6.00 (±2.28)	6.38 (±2.38)	6.32 (±1.87)	6.81 (±1.39)	6.80 (±1.56)
Basil	4.49 (±2.52)	4.91 (±2.26)	4.80 (±2.02)	6.10 (±2.01)	5.88 (±2.20)	5.89 (±2.17)	6.89 (±1.92)	6.61 (±1.91)	6.52 (±2.02)
Violet flower	5.66 (±2.03)	5.05 (±1.86)	5.66 (±1.50)	3.67 (±2.20)	4.06 (±1.75)	4.33 (±1.89)	3.42 (±2.44)	5.11 (±1.90)	5.47 (±1.60)
Fig flower	4.34 (±2.40)	3.49 (±1.72)	4.82 (±1.83)	4.37 (±2.58)	4.90 (±1.74)	5.06 (±1.96)	6.13 (±1.61)	6.76 (±1.66)	6.72 (±1.67)
Cake	5.23 (±2.88)	5.40 (±3.13)	5.40 (±2.84)	5.79 (±2.81)	6.55 (±2.64)	6.00 (±2.62)	6.72 (±1.73)	7.39 (±1.62)	7.44 (±1.79)
Peach	6.88 (±1.69)	6.00 (±2.02)	6.01 (±2.19)	5.57 (±2.31)	5.72 (±2.26)	4.89 (±2.07)	5.81 (±2.03)	5.95 (±1.96)	5.90 (±1.73)
Incense	3.21 (±1.95)	3.48 (±1.49)	3.62 (±1.82)	5.38 (±2.38)	5.52 (±2.14)	4.50 (±2.39)	7.26 (±1.74)	6.84 (±1.72)	6.00 (±1.72)
Strawberry	7.19 (±2.07)	6.30 (±2.02)	7.38 (±1.44)	5.78 (±2.55)	5.99 (±1.76)	6.56 (±2.14)	6.64 (±1.68)	6.70 (±1.68)	6.53 (±1.35)
Lilac	6.18 (±2.88)	6.69 (±2.20)	7.17 (±1.53)	6.60 (±2.28)	6.88 (±1.99)	6.81 (±1.84)	6.39 (±2.11)	6.23 (±1.74)	6.53 (±2.05)
Lichen	4.81 (±2.06)	4.36 (±1.68)	4.23 (±1.64)	4.79 (±2.67)	4.44 (±2.04)	4.18 (±1.50)	5.06 (±2.01)	5.67 (±1.80)	5.26 (±2.62)
Mango	5.52 (±2.63)	4.89 (±1.92)	5.25 (±1.23)	5.33 (±2.61)	5.22 (±2.19)	5.11 (±2.03)	6.37 (±1.60)	6.88 (±1.66)	6.47 (±1.76)
Chocolate	5.80 (±2.48)	5.21 (±2.24)	5.65 (±1.87)	5.42 (±2.34)	5.75 (±2.46)	5.05 (±2.50)	3.76 (±2.33)	4.35 (±2.14)	4.16 (±2.34)
Nakhla tobacco	6.95 (±2.38)	6.54 (±1.78)	6.76 (±2.22)	5.88 (±2.24)	6.32 (±1.54)	6.55 (±2.29)	5.97 (±1.75)	6.24 (±1.70)	6.24 (±1.81)
Rosemary	5.07 (±2.37)	5.35 (±1.99)	5.27 (±2.28)	7.19 (±2.37)	6.81 (±1.79)	6.33 (±2.05)	7.54 (±1.44)	7.29 (±1.33)	6.99 (±1.57)
Detergent	5.68 (±2.45)	5.54 (±2.97)	5.73 (±2.44)	8.09 (±1.52)	7.70 (±2.10)	7.81 (±1.51)	6.72 (±1.26)	7.29 (±1.34)	6.45 (±1.51)
Leather	2.62 (±1.68)	2.31 (±1.44)	1.99 (±1.54)	3.44 (±2.55)	3.72 (±2.43)	2.95 (±1.92)	6.19 (±2.35)	7.07 (±1.80)	6.55 (±1.92)

*Note*. Standard deviations are presented in parentheses.

#### Procedure

The present experiment was divided into two parts, separated by 1 week. The time of day of the experimental session was unchanged for a given participant.

#### First session

First, participants were requested to rate the pleasantness, familiarity, and intensity of the twenty target odorants. Like all the ratings they had to do, these were realized on continuous scales presented on a computer screen. Participants had to move a vertical marker with the mouse across a horizontal line and click to indicate their rating. Participants judged the pleasantness of the odor from *very unpleasant* (left on the scale = 1) to *very pleasant* (right on the scale = 10); the subjective familiarity from *not familiar at all* (left) to *very familiar* (right); and the subjective intensity from *not perceived* (left) to *very strong* (right).

Second, participants were given 12 coins of 5 CHF (approximately 12×5$). They were told that they were going to smell 20 smells, and that they should decide *before* exposure to a given smell, whether they were going to pay nothing or 5 CHF for it. They were further informed that in total, they had to pay for half of the odors (10 out of 20). Since these choices were made before knowing the odor on a given trial, the decision that they took cannot be related to any pre-existing preference for the odor. They were instructed to make a genuine choice before each trial, and not to use strategies, such as for instance giving 5 CHF for the ten first odors, and then nothing, or alternate between nothing and 5 CHF during each trial. For each trial, once they had made up their mind and paid 0 or 5 CHF, they were exposed to the odor. During this choice phase, participants were presented with two kinds of odors: (a) ten odors that they had rated as unpleasant (i.e., for one half of the trials; mean pleasantness rating = 3.35±0.86 on a 10-point subjective scale), and (b) ten odors that they had rated as pleasant (for one half of the trials; mean pleasantness rating = 7.08±1.02 on a 10-point subjective scale described in the following paragraph). This procedure of choosing, possibly paying, and smelling was repeated for 20 trials.

In the next phase, participants were presented with all 20 odors one more time, and rated each for pleasantness, familiarity and intensity again.

Finally, participants were presented with the twenty already presented target odors, together with ten new odors (distractors) and were requested to indicate whether they had already smelled each odor during the experiment. For each odor, if they answered yes, they were then asked to specify if they had designated this odor as chosen or rejected during the choice phase. Before being asked to do so at the end of the experiment, participants were not aware that they would have to complete this memory task. During the entire experiment, the order in which odors or pairs of odors were presented was controlled. For each presentation of an odor, participants were instructed to smell the odor during two inspirations at most.

#### Second session 1 week later

Participants were requested to rate the pleasantness, familiarity, and intensity of the twenty odorants.

#### Data analyses

In the free-choice paradigm, cognitive dissonance is assumed to arise while choosing between difficult pairs, i.e. pairs containing similarly liked stimuli [6]. In our new paradigm, we expected cognitive dissonance to occur for unpleasant odors, but not for pleasant ones. As described above, our rationale was that spending money is normally done only for desired outcomes. So spending money and receiving a negative outcome would cause dissonance. This should not occur for positive outcomes. The analyses reported here were consequently conducted on the half of odors initially rated most unpleasant, on an individual basis.

### Results

#### Choice-induced changes of preferences for odors

**Figure 3:**
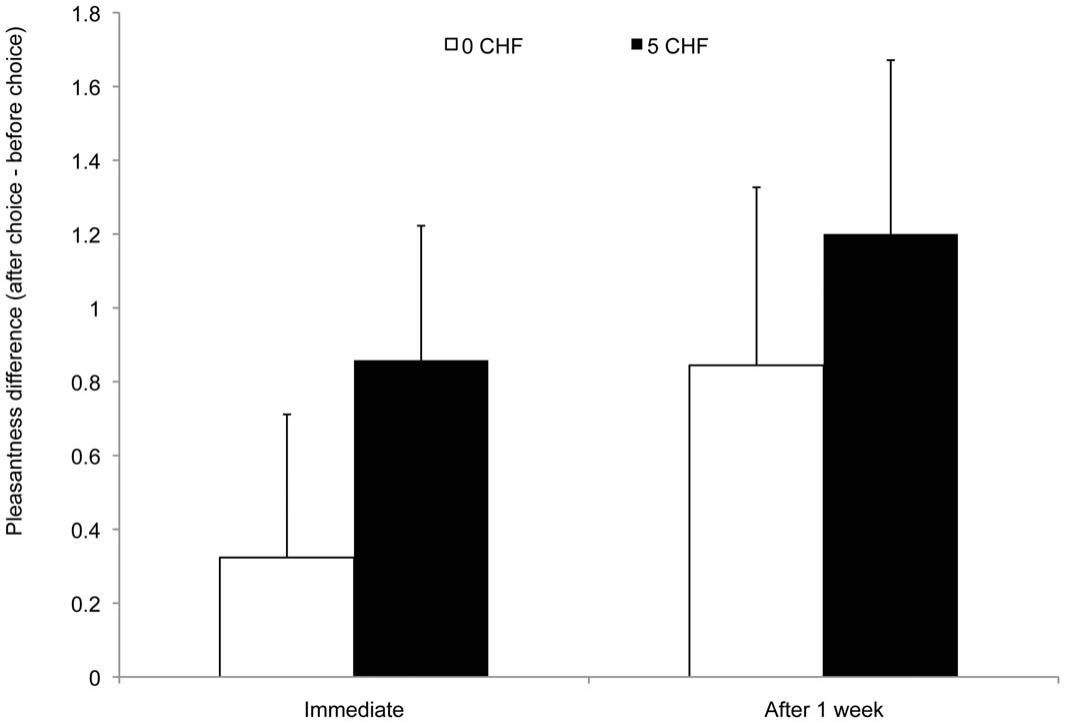
Pleasantness rating modulation for unpleasant odors following monetary choice in Experiment 3. Choice was between spending no money (0 CHF) vs. 5 CHF (approximately 5$) to be delivered, unknowingly, with unpleasant odors. The bars on the left represent the difference between pleasantness ratings before and just after choosing between pairs of odors. The bars on the right represent the difference between pleasantness ratings before and 1 week after these choices. Error bars represent the standard error to the mean.

The repeated measures ANOVA with the factor choice (0 CHF, 5 CHF) performed on the difference between pleasantness prechoice and postchoice ratings was significant [*F*(1,22) = 7.67, *p* = .011, η^2^ = .26]. Unpleasant odors on which participants chose to spend 5 CHF were evaluated as significantly more pleasant than odors for which participants did not spend money on (see Figure 3).

##### Choice-induced changes of preferences for odors during the second session (after 1 week)

A repeated measures ANOVA with the factor choice (chosen, rejected) performed on the difference between prechoice and postchoice ratings was significant [*F*(1,22) = 6.72, *p* = .017, η^2^ = .23]. Unpleasant odors on which participants chose to spend 5 CHF were evaluated as significantly more pleasant one week later than odors for which participants did not spend money on.

The repeated measures ANOVA with the factors period (immediately after choice, 1 week after choice) and choice (0 CHF, 5 CHF) performed on the difference between prechoice and postchoice ratings revealed no significant interaction between these factors [*F*(1,22) = 17.50, *p*<.001, η^2^ = .44]. Taken together, these results indicate that pleasantness ratings changes were not statistically different immediately after and 1 week after the choice, which suggests stability over time.

##### Memory performances

Participants remembered and well discriminated the presented odors from the distracting odors (mean hit rate: HR = 0.86, d’L = 3.33, CL = −0.38). Memory performance for the choice was not significantly above chance (HR = 0.49, *t*(22) = −0.45, *p* = .657).

##### Familiarity and intensity ratings

The mean familiarity and intensity ratings for all the odors are reported in Table 2.

Familiarity ratings between odors for which participants spent 0 vs. 5 CHF were neither significantly different just after the first choice [*F*(1,22) = 1.14, p = .297] nor 1 week after the first choice [*F*(1,22) = 1.38, p = .253]. Likewise, intensity ratings between odors for which participants spent 0 vs. 5 CHF were neither significantly different just after the first choice [*F*(1,22) = 0.59, p = .451], nor 1 week after the first choice [*F*(1,22 = 0.43, p = .518].

### Discussion

The data show that olfactory preferences can be modulated by a monetary choice across long time spans. Both 10 min and 1 week after a difficult choice, unpleasant odors on which participants had spent 5 CHF, were evaluated more positively compared to a first pleasantness evaluation than odors participants did not spend money on.

Crucially, participants were not presented with the odor prior to their payment choice. Their choice cannot consequently reveal their pre-existing preferences. This suggests that the results obtained in Experiments 1 and 2 are robust to settings in which Chen and Risen’s [34] valid critique does not apply. The results that choice has a modulatory impact on preferences and that this modulation is long-lasting are therefore more than a mere artifact of the free choice paradigm.

The result that preference modulation after difficult choice in Experiments 1 and 2 did not require an explicit memory of the choice is not directly discussed by Chen and Risen [34]. Nevertheless due to the inclusion of choice memory as a variable, the design of Experiment 3 in principle also permits a validation of this result. However, as the memory of the choice was at random level in Experiment 3, we could not conclusively interpret the trials that were correctly remembered as something other than chance. In particular, this means it would be misleading to split the data into remembered vs. forgotten trials, in order to analyze differences along this dimension. If anything, it seems to indicate that choice-induced preference modulation did not require an explicit memory of the choice to occur.

## General Discussion

The two first experiments of this manuscript have shown that after a choice between two odors that are perceived as equally pleasant, pleasantness ratings for these odors were changed in the long run. The second experiment shows that it holds true even when the choice made was not explicitly remembered. Results therefore suggest not only that pleasantness ratings change following a difficult choice remains stable for at least 1 week, but also that this stability can take place even when the choice has been forgotten. The third experiment addresses a potential flaw of the free-choice paradigm and shows that the fundamental results hold even when Chen and Risen’s [34] valid point, i.e. pre-existing preferences could explain post-choice preference modulation, is taken into account.

### Experienced Utility Versus Predicted Utility

In these three studies, participants were asked to rate the pleasantness of actually presented odors. Thus, along with the results of Coppin and colleagues [7], our findings extend results found in measuring predicted utility (e.g., [49]), where participants had to imagine how pleasant it would be to go to a given vacation destination) by showing that postchoice preference changes can also be found in experienced utility.

### Long Term for Both Chosen and Rejected Stimuli

The results from our two first experiments, obtained using a variant of the free-choice paradigm, suggest that olfactory pleasantness ratings can be modulated by a difficult choice in long time spans. Both 10 min and 1 week after a difficult choice, chosen odors were evaluated more positively (Experiments 1 and 2) and rejected odors were evaluated less positively (Experiment 1) compared with a first pleasantness evaluation.

### Impact of Choice on Preferences

In Experiment 3, a design derived from the effort-justification paradigm [48] was used. More precisely, participants were making choices about odors *before* smelling them. In this context, choice cannot be a reflection of pre-existing preferences. The issue raised by Chen and Risen [34], i.e. measured postchoice preference change may not necessarily be driven by an influence of choice on preferences, but could be due to the fact that choices reveal information about pre-existing preferences, cannot consequently apply. Yet, postchoice preference modulation was still reliably shown and demonstrated to be stable over time. The results from this third experiment are interesting for two reasons. First, they are an important complement to Experiments 1 and 2, as they allow concluding unequivocally that choices does have a modulatory influence on preferences, and that this influence is long-lasting. Second, they contribute to the current debate regarding the influence of choice on preferences (e.g., [33]–[38], [40]). Getting around the issue raised by Chen and Risen [34] when studying the impact of choice on preference now appears as crucial for future work. Risen and Chen [41] have mentioned four potential promising strategies in this respect. In “*removing the information from choice*” (the third strategy proposed by Risen & Chen [41]), the design of our third experiment, i.e. using a choice phase *before* the actual presentation of the stimuli on which a choice is made, proves to be potentially promising for future work.

### Choice Coherence

In Experiments 1 and 2, choice coherence–the likelihood of making a second choice similar to the first one–was high, even after 1 week. Such a result has a limited impact because we cannot exclude that this high choice coherence is based on a preexisting difference in pleasantness between chosen and rejected odors before the second choice. In other words, following the first choice phase, chosen odors were on average rated as much more pleasant than the rejected ones. It could have made the second choice an “easy” one, mainly driven by this pleasantness difference between the paired odors.

### Implicit Long-Term Stability

A growing body of research has explored the implicit mechanisms underlying preference modulation by choice (e.g., [35], [40], [49], [50]) and when the first choice is not explicitly remembered [7], [18].

Aldrovandi and Heussen [51] argued that “although memory is clearly involved in the processes of judgments and decision, memory cannot be a good candidate to provide the stability of preferences” (p. 2–3). In terms of psychological mechanisms, the result that postchoice preference modulation does not require an explicit memory of choice seems to challenge traditional accounts based on cognitive dissonance reduction, which emphasize cognitive accessibility [11], [14]–[16]. This conscious form of cognitive dissonance reduction can consequently be excluded as the only determinant of the preference modulation that occurred in the studies we presented here. However, if, as suggested notably by Jarcho and colleagues [13], cognitive dissonance reduction could be achieved in an unconscious fashion, then our results could be interpreted as evidence for such a mechanism. This would imply that different drivers of preference modulation may operate at the same time at different levels of processing. The differentiation and consolidation theory [52], [53] also suggests that postdecision differentiation (also called consolidation) processes, leading to a perceived congruency between a decision and current attitudes, may be unconscious [52].

### Conclusion

Our results have implications for a link between flexibility and stability of preferences: The current findings reveal how arbitrary-seeming momentary decisions can have a surprisingly *long-term* effect on preferences, and that this is true *even if the triggering decisions are forgotten.* The asymmetry between the fleetingness of the cause and the long-lastingness of its effect is particularly striking because it can operate independently from conscious recollection.
